# The Role of an Educational Program in Reducing Symptom Severity in Women with High Risk for Carpal Tunnel Syndrome

**DOI:** 10.3390/medsci13030094

**Published:** 2025-07-22

**Authors:** Amira Elhoufey

**Affiliations:** Department of Community Health Nursing, Alddrab University College, Jazan University, Jazan 45142, Saudi Arabia; aelhoufey@jazanu.edu.sa

**Keywords:** carpal tunnel syndrome, educational program, women, functional status, intervention

## Abstract

**Aim:** This study aimed to assess the effect of educational programs on symptom severity for women at high risk of carpal tunnel syndrome (CTS). **Methods**: A quasi-experimental design was applied. A purposive sample of 250 women at high risk of CTS was selected from the Faculty of Nursing, Assiut University, Egypt. Data collection instruments included a structured interview questionnaire and the Boston Carpal Tunnel Syndrome Questionnaire (BCTQ). **Results**: Most participants were middle-aged (41–50 years), married, and had higher education. At baseline, 61.2% of participants reported mild symptoms, 24.8% moderate, and 11.6% were asymptomatic. Following the educational program, symptom severity was significantly improved (*p* = 0.007). The proportion of asymptomatic participants increased from 11.6% to 20.4%, while those with moderate symptoms decreased from 24.8% to 6.4%. Functional status also improved significantly, with the percentage of participants reporting no difficulty increasing from 17.6% to 30% (*p* = 0.008). We found a significant reduction in symptom severity scores (*p* = 0.05) and functional impairment (*p* = 0.008). **Conclusions:** The educational program effectively reduced CTS symptoms and improved hand function, demonstrating its potential as a preventive and therapeutic intervention for women at high risk of CTS. However, this study’s quasi-experimental design without a control group and a short follow-up period limits conclusions regarding long-term effectiveness and causal inference.

## 1. Introduction

Technology has become an essential component of modern daily life, encompassing smartphones, computers, and tablets. Virtually everyone is connected to some form of technology, a trend that also extends to healthcare professionals. Patient data and clinical information are increasingly accessed and managed through digital platforms. However, the widespread use of technology is not without adverse effects. Repetitive motions associated with prolonged device usage have been linked to a higher risk of developing musculoskeletal disorders, such as carpal tunnel syndrome (CTS) [1]. CTS is a musculoskeletal disorder characterized by anterior wrist pain, hand paresthesia, and muscular weakness. Epidemiological data indicate an increasing prevalence in China, especially among middle-aged and older women, as well as individuals with a history of repetitive strain injuries or occupational exposure. Without timely and appropriate intervention, CTS may progress to complications such as thenar muscle atrophy, which can significantly impair quality of life [2]. The risk of developing CTS increases to about 10% among individuals engaged in repetitive manual work, especially tasks involving frequent flexion and extension of the hands. Office workers, especially women over the age of 30, are also considered at risk for wrist and hand disorders. Nonetheless, CTS is not formally recognized as an occupational disease among office workers despite its association with repetitive hand movements and prolonged computer use [3]. Early detection and treatment are critical to prevent permanent nerve and muscle damage. Educating patients on ergonomic practices and lifestyle changes plays a vital role in managing symptoms and minimizing the risk of recurrence. Physical therapists use evidence-based and individualized approaches to help patients achieve long-term symptom relief and improve their quality of life. Tailored educational programs are recommended to address each patient’s specific needs and support functional recovery and overall health [4]. Physiotherapy is the primary non-invasive treatment modality for individuals with mild to moderate CTS. Standard interventions encompass therapeutic ultrasound, electrotherapy, low-level laser therapy, magnetotherapy, and kinesitherapy, which involve structured movement-based rehabilitation. Stretching and nerve mobilization exercises for the median nerve are considered the most effective. However, there is limited research on the success of exercise-only therapy, with most studies focusing on patients already diagnosed with CTS rather than those at risk or in the early stages of symptoms [5]. Nurses play an essential role in the assessment and implementation of targeted interventions aimed at mitigating pain and enhancing functional outcomes in individuals affected by CTS. They educate patients on workplace ergonomics, advising on proper hand, wrist, and forearm positioning. Ergonomic recommendations for individuals with CTS include maintaining forearms parallel to the floor, keeping wrists in a neutral position, and ensuring elbows are relaxed at approximately a 90-degree angle. Nurses are responsible for reinforcing these postural guidelines and encouraging patients to regularly alternate hand positions, particularly during extended tasks such as driving or mobile device use, and to adopt a light touch when typing to minimize strain on the median nerve. Regular breaks are encouraged to allow stretching and muscle relaxation [6].

CTS significantly affects individuals’ quality of life, is highly prevalent, and burdens healthcare systems financially. Nurses are pivotal in teaching proper body mechanics, good posture during work and daily activities, and specific exercises to treat and prevent CTS symptoms [7].

### Research Hypotheses

It is hypothesized that women at high risk for CTS who participate in the educational program will exhibit a significant reduction in symptom severity. It is also hypothesized that those receiving the intervention will demonstrate statistically significant improvements in hand functional status.

Due to the paucity of research concerning risk factors for CTS among women in Assiut, this study seeks to evaluate the effectiveness of an educational program for women at high risk of developing CTS. This study aimed to evaluate the effect of an educational program on symptom severity among women at high risk for CTS.

## 2. Methods

Research Design: A quasi-experimental pre-test/post-test design was employed to evaluate the impact of an educational program on symptom severity and functional status among women at high risk for CTS. This approach was selected without a control group due to feasibility constraints and ethical considerations related to withholding potentially beneficial education.

### 2.1. Subjects

Based on Cohen’s guidelines for effect size and assuming a medium effect size (f = 0.25), an alpha level of 0.05, and a power (1–β) of 0.80, the required sample size would be approximately 100–150 participants. By selecting 250 participants, this study exceeded this threshold, increasing the likelihood of detecting true effects and allowing for subgroup analyses (e.g., by age group, BMI (kg/m^2^), or occupational exposure).

A total of 250 women were recruited from the Faculty of Nursing at Assiut University. Participants were selected based on inclusion criteria that included being between the ages of 20 and 60 years, at high risk for CTS, engaged in job duties involving regular computer use, and verbally communicative, mentally alert, and capable of following instructions.

### 2.2. Data Collection Tools

Two Primary Tools Were Used to Collect Data for This Study

Tool I: Patient Interview Questionnaire: This structured questionnaire consisted of three main parts. The first part collected sociodemographic data, including participants’ age, marital status, place of residence, and level of education. The second part focused on risk factors for CTS and included information on medical history such as chronic diseases, previous surgeries, hospital admissions, and allergies. It also included obstetric history covering pregnancy status, contraceptive use, and menopausal status. Additionally, it assessed work-related organizational variables by asking participants to respond “yes” or “no” to nine questions [8]. These questions included the following:

1. Do you work overtime?
2. Do you have a second job?
3. Do you hold your hand in one position repetitively?
4. Do you perform rapid “trigger” finger movements?
5. Do you engage in repetitive pressing with your hands?
6. Do you frequently flex and extend your wrist for more than two-thirds of your working time?
7. Do you frequently perform sustained forceful hand motions?
8. Do you often bend and twist your hands or wrists for 3.5–6 h daily?
9. Do you use a computer mouse for more than 20 h per week?

Each “yes” response was assigned one point, and a higher total score indicated an increased risk of developing CTS.

Tool II: Functional Status and Symptom Severity Questionnaire: This self-administered instrument [7] included two scales: The Symptom Severity Scale (SSS) and the Functional Status Scale (FSS). The SSS comprised 11 items assessing six major symptom domains: pain, numbness, paresthesia, nocturnal symptoms, weakness, and overall functional impairment. The FSS evaluated eight activities commonly affected by CTS, including writing, buttoning clothes, holding books while reading, gripping a telephone handle, carrying a grocery basket, performing household chores, opening jars, and bathing and dressing. The questionnaire was administered twice—once after obtaining informed consent and again after completion of the educational program—with each administration taking approximately 30 min.

### 2.3. Scoring System

Symptom Severity Scale (SSS) (Table 1): Each item was rated on a 5-point Likert scale (1 = no symptoms, 5 = most severe symptoms). Functional Status Scale (FSS) (Table 2): Each activity was rated from 1 (easiest) to 5 (most difficult), with the mean of the eight items calculated for the final score. Higher scores on both scales indicated worse symptoms and greater functional impairment.

#### Validity and Reliability of the Tools

Content validity of the tools was established through review by a panel of five experts in community health nursing at Assiut University. Based on their feedback, minor revisions were made to enhance clarity and precision. Reliability was assessed using Cronbach’s alpha coefficient. The structured interview questionnaire showed high internal consistency with a Cronbach’s alpha of 0.89. The Boston Carpal Tunnel Questionnaire (BCTQ) demonstrated strong test-retest reliability with a coefficient of 0.95, indicating excellent temporal stability.

### 2.4. Administrative Phase

An official permission letter was obtained from the Faculty of Nursing at Assiut University after providing a detailed explanation of the study’s purpose and objectives.

#### 2.4.1. Pilot Study

A pilot study was conducted with ten participants who met the inclusion criteria. The aim of the pilot was to assess the clarity, applicability, and feasibility of the study instruments. Minor modifications were made based on feedback before beginning the main data collection phase.

#### 2.4.2. Data Collection Phase

Ethical approval was obtained from the Ethics Committee of the Faculty of Nursing, Assiut University (IRB 12-2023). Face-to-face recruitment was conducted, and informed verbal consent was obtained after explaining the study’s objectives. Participants were assured of their right to withdraw at any time without penalty. Confidentiality and anonymity were strictly maintained throughout the study.

#### 2.4.3. Intervention Program Phases

Phase I: Preparatory and Planning Phase

A comprehensive literature review was carried out to understand relevant variables and guide protocol development. Site visits were conducted to prepare for participant recruitment, data collection logistics, and intervention delivery.

Phase II: Implementation Phase

Data collection and intervention implementation occurred over a period of three months from October to December 2024. Baseline assessments were conducted using Tool I (approximately 15 min) and Tool II (approximately 30 min) after explaining the study’s purpose. Participants received an instructional pamphlet written in simple Arabic and illustrated with photographs. The pamphlet covered information about CTS, warm-up exercises, wrist flexor and extensor stretches. The educational program was structured into two components and delivered using a standardized approach. All sessions were conducted using a pre-developed script, multimedia tools (videos and posters), and illustrated pamphlets to ensure consistency across participants. Each participant received a copy of the exercise guidebook in Arabic for reference at home. The program was facilitated by a certified community health nurse with expertise in occupational health and musculoskeletal injury prevention, who followed a standardized protocol throughout all sessions (See Supplementary file and Table S1). In Part I, two theoretical sessions lasting 30 min each were delivered using media aids such as videos and posters. These sessions covered the study’s aims, the importance of hand exercises in preventing computer-related disorders, and basic ergonomic and health tips. In Part II, participants were involved in live demonstrations and re-demonstrations of the prescribed exercises under the supervision of the instructor. This hands-on session ensured correct form and technique. To maintain fidelity, a session checklist was completed after each session to verify that all core components were delivered. The details of the exercise regimen included the following:

A. Warm-up: Five minutes of wrist mobility exercises.
B. Stretching and Strengthening Routine:
  - Wrist Flexor Stretch: Extend the arm and palm down, bend the wrist so the fingers point downward, and gently pull the hand toward the body.
  - Wrist Extensor Stretch: Extend the arm, bend the wrist, and gently pull the hand backward.
  - Medial Nerve Glide Exercise: A sequence of six hand positions involving wrist and finger movements, each held for 5 s and repeated 3–5 times.

Participants were instructed to perform the exercises once daily and to stop if pain or excessive fatigue occurred. Each stretch was held for 15 s.

Phase III: Evaluation Phase

One month after completing the intervention, the BCTQ was re-administered to evaluate changes in symptom severity and functional status compared to baseline measurements.

### 2.5. Data Analysis

The collected data were coded, entered, and analyzed using the Statistical Package for the Social Sciences (SPSS), version 22. Descriptive statistics (such as frequencies, percentages, means, and standard deviations) were used to summarize the study sample’s sociodemographic characteristics, risk factors, and baseline findings.

Inferential statistics were employed to examine the effect of the educational program on the severity of CTS symptoms and hand functional status. For continuous variables such as symptom severity and functional status scores, paired *t*-tests were used to compare pre- and post-intervention means. For categorical variables (e.g., symptom severity categories), McNemar’s test was used for binary outcomes, and the Stuart–Maxwell test was applied for multi-category outcomes to account for the paired nature of the data. A *p*-value of less than 0.05 was considered statistically significant.

## 3. Results

A total of 250 women at high risk for CTS participated in this study. Sociodemographic data (Table 3) revealed that the majority of participants were in the 41–50-year age group (n = 106, 42.4%), followed by those aged 31–40 years (n = 61, 24.4%). Most participants had completed university-level education (n = 173, 69.2%) and resided in urban areas (n = 187, 74.8%). A significant proportion of the sample was married (n = 207, 82.0%). Regarding medical history (Table 3), 114 women (45.6%) reported having osteoarthritis, 87 (34.8%) had diabetes mellitus, and 76 (30.4%) had hypertension. Additionally, 36 women (14.4%) reported a prior diagnosis of nerve compression, while 28 (11.2%) had a history of hand fracture. The Body mass index (BMI; kg/m^2^) assessment indicated that 126 participants (50.4%) were obese and 99 (39.6%) were overweight, with a mean BMI of 2.48 ± 0.34 kg/m^2^. Table 3 shows the obstetric history; 187 women (74.8%) had three to four pregnancies, and 103 (41.2%) were postmenopausal. Approximately 39.6% (n = 99) were currently using hormonal contraceptives. All participants engaged in repetitive hand positioning during work tasks (n = 250, 100%), and all used a computer mouse for more than 20 h per week (n = 250, 100%). Furthermore, 201 women (80.4%) worked overtime, and 202 (80.8%) performed frequent wrist flexion and extension movements for more than two-thirds of their working time (Table 4). Occupational exposure variables yielded a mean score of 4.96 ± 0.38, indicating substantial ergonomic risk among the study population. Regarding adherence to home exercises, we collected self-reported compliance data through structured interviews during the follow-up session. Of the 250 participants, 216 (86.4%) reported performing the prescribed exercises regularly (≥5 days/week), while 34 (13.6%) admitted to partial or inconsistent adherence. Symptom severity was assessed using BCTQ before and after the educational intervention. At baseline, 62 participants (24.8%) exhibited moderate symptoms, while 153 (61.2%) had mild symptoms, and 29 (11.6%) were asymptomatic. Following the implementation of the educational program, there was a statistically significant improvement in symptom severity (*p* = 0.001), with 51 participants (20.4%) becoming asymptomatic and 183 (73.2%) reporting only mild symptoms. No participants remained in the severe or very severe categories post-intervention (Table 5). Functional status also improved significantly, as evidenced by a shift from moderate difficulty in performing daily activities at baseline (n = 75, 30%) to only 19 (7.6%) post-intervention (χ^2^ = 25.12, *p* = 0.008) (Table 6). The number of participants reporting no difficulty increased from 44 (17.6%) to 75 (30%). Our results showed a significant decrease in the mean symptom severity score from 43.77 ± 4.30 pre-intervention to 23.77 ± 2.55 post-intervention (*p <* 0.05). Similarly, functional status scores markedly improved, decreasing from 27.72 ± 3.50 to 19.77 ± 1.88 (*p* = 0.008) (Table 6). These results demonstrate the effectiveness of the structured educational program in reducing both symptom burden and functional disability among women at high risk for CTS. A multiple linear regression model was conducted to identify significant predictors of symptom severity among women at high risk for CTS (Table 7). Statistically significant predictors included history of hand fracture (β = 0.388, *p* < 0.001) and menopausal status (β = 0.281, *p* < 0.001), both of which were positively associated with higher symptom severity scores. Higher educational levels showed a strong inverse relationship with symptom severity (β = −0.367, *p* = 0.001). Participation in the educational program showed a strong negative association with symptom severity (β = −0.467, *p* < 0.001), indicating a substantial reduction in symptoms post-intervention. Other variables such as diabetes, arthritis, and work-related exposure did not show statistically significant associations.

## 4. Discussion

Effective management of CTS necessitates a comprehensive, multimodal approach to alleviate symptoms and enhance wrist function. Physical therapists play a pivotal role by tailoring individualized treatment plans based on the severity of symptoms, patient-specific needs, and any existing contraindications. Early diagnosis and timely intervention are critical to prevent irreversible damage to the median nerve and associated musculature. Physical therapy interventions commonly include targeted therapeutic exercises, manual therapy techniques, and adjunct modalities such as electrotherapy. These strategies improve muscle strength, restore coordination, and reduce pain and sensory disturbances [9]. This study aimed to assess the effect of educational programs on symptom severity for women at high risk of CTS.

Regarding prior hand fractures, the present findings indicate that 11.2% of participants had a history of hand fractures. This aligns with previous research by Toth et al. [10] who reported that over one-quarter of Swedish women aged 55–90 years presenting with fragility fractures also had a concomitant history of hand fractures, which may predispose individuals to structural changes or biomechanical alterations contributing to CTS development [10].

Concerning BMI, the current results demonstrated that half of the participants were classified as obese. This contrasts with findings from Alduraibi et al. [11], who reported that more than half of their sample population had normal BMI values. Nevertheless, obesity remains a well-established risk factor for CTS due to increased mechanical pressure on the median nerve secondary to fat deposition and fluid retention within the carpal tunnel.

This study found that 41.2% of participants were postmenopausal. The decline in estrogen levels during menopause may contribute to fluid retention and weight gain, thereby increasing intracarpal pressure and compressing the median nerve. This observation is consistent with Rotaru-Zavaleanu et al. [12], who identified menopause as a significant risk factor for the development of occupational-related CTS.

Baseline assessment revealed that approximately one-quarter of the sample exhibited moderate symptom severity. This finding corresponds with Abdel-Fattah et al. [13], who reported that 26.7% of their cohort presented with moderate symptom severity.

This study demonstrated a statistically significant improvement in hand functional status after the educational intervention. This may be attributed to the high educational level of participants, which could enhance adherence to health education. The result of this study agreed with previous work by Łach and Cygańska [5], which showed improvement in hand function among the studied sample after applying the exercise protocol.

Analysis of predictive variables revealed that age, diabetes, arthritis, history of fractures, menopause, and work-related factors were significant negative predictors of symptom severity. This finding may be due to jobs involving repetitive wrist and hand motions, such as typing or assembly line work, which are associated with higher CTS incidence. This is consistent with findings from Alduraibi et al. [11], who identified age, chronic comorbidities, and employment type as major determinants of CTS risk.

The findings of this study indicate a significant association between age and the development of CTS, with working women aged over 45 years showing a higher likelihood of experiencing CTS symptoms. This finding may be due to the association with age-related histopathological alterations in the transverse carpal ligament, which may reduce its elasticity and increase intracarpal pressure, thereby predisposing individuals to CTS. This is supported by a previous study [14], which revealed that increased age increases the risk of many health problems, such as CTS.

This study has some limitations. First, the quasi-experimental design without a control group limits the ability to establish a causal relationship between the educational intervention and observed improvements in symptom severity and functional status. Second, the reliance on self-reported data may introduce potential recall and response biases, particularly regarding medical and occupational histories. Third, the sample was drawn from a single institution in Egypt, which may limit the generalizability of the results to broader populations or different occupational settings. Fourth, while the follow-up period demonstrated short-term benefits of the educational program, longer-term outcomes beyond one-month post-intervention were not assessed, limiting conclusions about the sustainability of the observed improvements. Given that behavioral change often requires reinforcement over time, future studies should incorporate extended follow-up periods and consider booster sessions to evaluate long-term adherence and maintenance of symptom reduction. Finally, although efforts were made to control confounding variables, residual confounding due to unmeasured factors cannot be ruled out.

## 5. Conclusions

The findings of this study demonstrated that the structured educational program significantly reduces symptom severity and improves functional status among women at high risk for CTS. Following the intervention, there was a notable reduction in both the severity of symptoms and the level of functional impairment, demonstrating the success of preventive health education in slowing the progression of CTS. Age over 45 years, presence of comorbidities such as diabetes and osteoarthritis, history of hand fractures, menopausal status, and occupational exposure to repetitive hand movements were identified as significant predictors of increased symptom severity. These results underscore the critical need for early identification of risk factors and the integration of focused educational initiatives into primary healthcare, particularly for women in the workforce who experience prolonged computer use. The results support the incorporation of ergonomic awareness programs and routine screening for early signs of CTS in high-risk populations to prevent long-term disability and improve quality of life.

## Figures and Tables

**Table 1:** Symptom Severity Scale scoring.

Severity Level	Score
Asymptomatic	11
Mild	12–22
Moderate	23–33
Severe	34–44
Very severe	>45

The Symptom Severity Scale (SSS) consists of 11 items rated on a 5-point Likert scale (1 = no symptoms, 5 = most severe). Total score ranges from 11 to 55.

**Table 2:** Functional Status Scale (FSS) scoring.

Functional Status	Score
No difficulty	1–8
Little	9–16
Moderate	17–24
Intense	25–32
Cannot perform the activity at all	>32

The Functional Status Scale (FSS) assesses eight daily activities affected by CTS. Each item is scored from 1 (easiest) to 5 (most difficult).

**Table 3:** Sociodemographic, Medical History, and Obstetric Risk Factors of Women at High Risk for CTS.

Variable	Number (250)	%
Age (years)		
20–30	44	17.6
31–40	61	24.4
41–50	106	42.4
>50	39	15.6
Education level		
Secondary	31	12.4
High	173	69.2
Post-graduate	46	18.4
Residence:		
Urban	187	74.8
Rural	63	25.2
Marital status:		
Married	207	82.0
Unmarried	43	18.0
History of chronic disease *:		
No	31	12.4
Hypertension	76	30.4
Diabetes	87	34.8
Osteoarthritis	114	45.6
Cancer	19	7.6
Renal disease	37	14.8
Peptic ulcer	13	5.2
Nerve compression:		
Yes	36	14.4
No	87	34.8
Don’t know	127	50.8
History of hand fracture		
Yes	28	11.2
No	222	88.8
Side of hand fracture: (n = 14)		
Right	14/28	50
Left	10/28	35.7
Both	4/28	14.3
Duration of fracture: (n = 14)		
<5 years	4/14	28.6
5–10 years	7/14	50
>10 years	3/14	21.4
History of Gravidity:		
No	24	9.6
1–2	31	12.4
3–4	187	74.8
>4	8	3.2
History of Parity:		
No	31	12.4
1–2	31	12.4
3–4	180	72
>4	8	3.2
Previous pregnancy complications:		
Yes	24	9.6
No	226	90.4
Menopause:		
Yes	103	41.2
No	147	58.8
Current Hormonal contraceptive uses:		
Yes	99	39.6
No	151	60.4

* Participants could report more than one chronic condition.

**Table 4:** Occupational and work-related risk factors among the studied women.

Work Variable	Number (250)	%
Do not work overtime	49	19.6
2. Work overtime	201	80.4
3. Do not have a 2nd job	208	83.2
4. Having a 2nd job	42	16.8
5. Repetitively holding hand in one position	250	100
6. Rapid “trigger” finger movements	211	84.4
7. Repetitive pressing with hands	173	69.2
8. Frequent wrist flexion and extension occurring for more than two-thirds of the work time.	202	80.8
9. Frequent, sustained, forceful hand or wrist movements during work tasks.	113	45.2
Prolonged bending and twisting of the hands or wrists, performed for approximately 3.5 to 6 h per day.	14	5.6
Use of a computer mouse exceeds 20 h per week.	250	100

**Table 5:** Comparison of symptom severity before and after implementation of the educational program.

	Before Program	After Program	*p*-Value
Severity Level			
- Asymptomatic	29 (11.6%)	51 (20.4%)	*p* < 0.001 *
- Mild	153 (61.2%)	183 (73.2%)	
- Moderate	62 (24.8%)	16 (6.4%)	
- Severe	6 (2.4%)	0 (0)	
- Very severe	0 (0)	0 (0)	
Hand Functional Status			
- No difficulty	44 (17.6%)	75 (30%)	*p* < 0.001 **
- Little	131 (52.4%)	156 (62.4%)	
- Moderate	75 (30%)	19 (7.6%)	
- Intense	0 (0)	0 (0)	

* Stuart–Maxwell test, ** McNemar test, statistically significant at *p* < 0.05.

**Table 6:** Mean scores of symptom severity and functional status pre- and post-educational intervention.

	Pre	Post	95% Confidence Intervals (CI)	*p* Value
Symptoms severity	43.77 ± 4.30	23.77 ± 2.55	19.86–20.14	<0.05 *
Functional status	27.72 ± 3.5	19.77 ± 1.88	7.65–8.25	0.008 *

* Paired *t*-test, statistically significant at *p* < 0.05.

**Table 7:** Predictors of Symptom Severity among Studied Women.

Variable	Unstandardized Coefficient (B)	St. Error	Standardized Coefficient (β)	t	*p*-Value	95% CI Lower Bound	95% CI Upper Bound
Constant	6.072	12.638	—	0.472	0.620	−18.701	30.975
Age	0.103	0.112	−0.047	0.934	0.324	−0.312	0.118
Education level	−0.434	0.845	−0.367	3.329	0.001 *	0.42	0.153
Diabetes	0.301	0.203	0.128	1.424	0.139	0.099	0.704
Arthritis	0.315	0.200	0.134	1.554	0.121	−0.083	0.612
History of fracture	1.675	9.411	0.388	5.234	0.001 *	0.624	0.287
Menopause	0.144	0.025	0.281	5.361	0.001 *	−0.843	−0.432
Work variables	−0.105	0.113	−0.049	−1.467	0.139	−0.099	0.701
Educational program	−0.559	0.063	−0.467	−8.923	0.001 *	−0.682	−0.433

Binary variables (e.g., menopause, fracture history) were coded as 0 = absence and 1 = presence of condition. Continuous variables (e.g., age, education level) were entered as numerical values. * Statistically significant at *p* < 0.05.

## Data Availability

Anonymized data are available upon reasonable request and with institutional approval.

## Supplementary Materials

The following supporting information can be downloaded at https://www.mdpi.com/article/10.3390/medsci13030094/s1: Table S1: Components of the Educational Program.
