# Supplementary material for: The Role of an Educational Program in Reducing Symptom Severity in Women with High Risk for Carpal Tunnel Syndrome
Source: Med Sci (Basel). 2025 Jul 22;13(3):94. doi: 10.3390/medsci13030094 (PMC12371919; doi:10.3390/medsci13030094)
Supplement: Supplementary file 1 [file medsci-13-00094-s001.zip › medsci-3706532-supplementary.pdf]

The Role of an Educational Program in Reducing Symptom Severity in Women with High Risk for Carpal Tunnel Syndrome

Supplementary material

Educational Program Content and Structure:

The educational program was designed based on evidence-based guidelines and tailored specifically for women at high risk for Carpal Tunnel Syndrome (CTS). The program aimed to improve awareness, promote ergonomic practices, and teach self-management strategies to prevent symptom progression. It consisted of two theoretical sessions and one practical session, each lasting approximately 30 minutes, delivered over a three-month period from October to December 2024.

The content was standardized across all participants and delivered using a structured format that included:

- Printed instructional pamphlets written in simple Arabic with illustrations.
- Multimedia tools, including videos and posters.
- Live demonstrations of exercises by both the instructor and participants.

Each participant received a copy of the instructional pamphlet containing step-by-step descriptions and photographs of the recommended exercises. Additionally, participants

were encouraged to ask questions during and after the sessions to ensure understanding and engagement.

Session Components Overview (See Supplemental Table 1):

1. Theoretical Component (Part I):
    - Two 30-minute sessions:
        - Session 1: Introduction to CTS, risk factors, and the importance of early intervention.
        - Session 2: Ergonomic principles, posture correction, and the role of physical activity in preventing CTS.
2. Practical Component (Part II):
    - One 30-minute session:
        - Live demonstration and guided performance of warm-up, stretching, and nerve gliding exercises.
        - Participants practiced under supervision to ensure correct form and technique.

Standardization and Fidelity Measures:

To ensure consistency and fidelity across all sessions, the following measures were implemented:

- All sessions were led by the same trained community health nurse, who holds a Master's degree in Community Health Nursing and has over five years of experience in occupational health education.
- A standardized script and visual aids (videos and posters) were used in every session.
- All participants received identical printed materials and participated in the same sequence of activities.
- A session checklist was used to document the delivery of all key components.
- At the end of each session, participants completed a brief comprehension quiz to assess their understanding of core concepts.
- During the follow-up phase, adherence to home exercises was assessed via structured interviews.

These measures ensured that the intervention was consistently delivered and that participants received uniform exposure to the educational content.

Instructor Qualifications:

The educational program was delivered by a certified community health nurse with expertise in preventive care and occupational health. The instructor had prior training in musculoskeletal injury prevention and had conducted similar educational interventions in workplace settings. Additionally, the program was developed in consultation with a panel of experts in nursing and ergonomics at Assiut University.

Instructor Qualifications

The educational program was delivered by a certified community health nurse holding a master's degree in Community Health Nursing and over five years of experience in occupational health education. The instructor had prior training in musculoskeletal injury prevention and had conducted similar educational interventions in workplace settings. The program was developed in consultation with a panel of experts in nursing and ergonomics at Assiut University.

Program Standardization Measures

To ensure consistency across all sessions:

- A standardized script was used for all theoretical and practical components.
- Identical visual aids (videos, posters, and illustrations) were shown to every participant.
- Each participant received the same illustrated exercise guidebook in Arabic.

- A checklist was completed after each session to confirm the delivery of all key points.
- A brief quiz was administered at the end of each session to assess understanding of core concepts.
- Adherence to home exercises was assessed via structured interviews during the follow-up phase.

Home Exercise Protocol

Participants were instructed to perform the following routine once daily:

- Warm-up: 5 minutes of wrist mobility exercises
- Wrist Flexor Stretch: Extend arm, palm down; gently pull fingers toward the body (15 seconds x 3 sets)
- Wrist Extensor Stretch: Extend arm, palm up; gently pull fingers backward (15 seconds x 3 sets)
- Median Nerve Glide Exercise: Sequence of six hand/wrist positions, each held for 5 seconds and repeated 3–5 times
- Total time: ~10–15 minutes/day
- Participants were advised to stop if they experienced pain or fatigue

Table S1: Components of the Educational Program

Component	Content Description	Duration	Delivery Method
Session 1: Theoretical	Introduction to CTS: Anatomy, symptoms, and risk factors; importance of early detection; consequences of untreated CTS	30 minutes	PowerPoint presentation, video, poster visuals
Session 2: Theoretical	Ergonomic principles: correct hand positioning, use of supportive devices, minimizing repetitive strain; lifestyle modifications	30 minutes	PowerPoint presentation, video, interactive discussion
Session 3: Practical	Warm-up exercises (5 min); wrist flexor stretch (15 sec x 3 reps); wrist extensor stretch (15 sec x 3 reps); median nerve glide exercise (sequence of 6 positions, 5 sec each x 3–5 reps); supervised practice and feedback	30 minutes	Live demonstration, participant re-demonstration, instructor feedback
Home Exercise Regimen	Daily routine: warm-up + stretching + nerve glides; duration: ~10–15 minutes/day; stop if pain/fatigue occurs	Self-directed	Illustrated pamphlet, verbal instructions
